# Identification of mRNAs Related to Tibial Cartilage Development of Yorkshire Piglets

**DOI:** 10.1155/2019/2365416

**Published:** 2019-11-05

**Authors:** Shuaifei Feng, Xiaoyong Du, Chao Wang, Dengdeng Ye, Guanjun Ma, Shuhong Zhao, Haiyan Wang, Xiangdong Liu

**Affiliations:** ^1^Key Laboratory of Animal Genetics, Breeding and Reproduction of Ministry of Education, College of Animal Sciences & Technology, Huazhong Agricultural University, Wuhan 430070, China; ^2^Hubei Key Laboratory of Agricultural Bioinformatics, College of Informatics, Huazhong Agricultural University, Wuhan 430070, China; ^3^Key Lab of Swine Healthy Breeding of Ministry of Agriculture and Rural Affairs, Guigang 537100, China

## Abstract

Cartilage dysplasia is one of the important reasons for the weakness of pig limbs and hooves. Porcine rickets with weak limbs and hooves bring huge economic losses to the pig industry. However, research on the development of pig cartilage is lacking. This study investigated the key genes and molecular mechanisms involved in cartilage development via an RNA-seq technique. Samples of proximal tibia cartilage were collected from three normal piglets with 1 day, 14 days, and 28 days of age, respectively, and then these samples were divided into two comparison groups (1-day vs. 14-day group, 14-day vs. 28-day group). Through the transcriptome analysis, 108 differentially expressed genes (DEGs), such as *FORL2*, were obtained from 1-day vs. 14-day comparison group, and 3602 DEGs were obtained from 14-day vs. 28-day comparison group, including *SOX9*, *BMP6*, and *MMP13*. The gene ontology (GO) functional and KEGG pathway enrichment revealed that many functions of DEGs were related to bone development. The pathways of DEGs from Day 1 vs. Day 14 were mainly enriched in mineral absorption, but the DEGs of Day 14 vs. Day 28 were enriched in osteoclast differentiation. Then, the expression patterns of six candidate genes were verified via qPCR. In conclusion, candidate genes affecting cartilage development in Yorkshire pigs were obtained by transcriptome analysis, and the clues showed that Day 14 to Day 28 is a more active and extensive period in cartilage developments, which played a key role in revealing the molecular mechanism of pig cartilage development basis, also compensating for vacancies in cartilage research.

## 1. Introduction

In the modern large-scale pig industry, pig's foot disease has become one of the most common and complex problems in pigs and occurs in pigs of all ages. Piglets are susceptible to sow injury or joint infection in the bed, causing foot and hoof disease, which affects the survival and growth rate of piglets during development. At present, leg and foot disease has become a major factor restricting the production, reproduction, and longevity of pigs, and it has also caused a large number of elimination and economic losses in the pig industry. Limb and foot disease is the second most important reason for the elimination of young sows [1]; Heinonen et al. have shown that sows eliminated by leg and foot disease account for 20–50% of all eliminated sows, and young sows are more susceptible to hoof rupture [2]. Therefore, further research on the pathogenesis and pathological mechanism of swine foot and hoof disease can help to improve limb and hoof problems and reduce economic losses.

It has been reported that osteochondrosis is an important cause of weakness in pigs' hooves [3]. Bone formation originated from mesenchymal progenitor cells, then undergoes development and differentiation into mature chondrocytes, and is eventually replaced by bone. Studies have reported that cartilage cells are differentiated in the agglutination center region of mesenchymal cells, and a cartilage primordium composed of immature circular chondrocytes is produced [4]. A number of transcriptional and signaling factors are involved in this process, in which the agglutination of mesenchymal cells can express an important transcription factor involved in chondrogenesis Sox9 [5].

The most common site of rickets is the knee joint [6], followed by the elbow joint, and the ankle and hip joints are relatively few [7]. Ytrehus and other studies have found that piglets' growth cartilage receives blood supply through the cartilage passage, but over time, the cartilage tube is converted to cartilage by softening, which is a physiological process that does not cause rickets disease. However, it was found that there is a correlation between an abnormal cartilage tube and rickets disease, which leads to local ischemic necrosis due to blood supply interruption, which eventually leads to secondary rickets [8]. Genetic factors play an important role in cartilage lesions. Studies have found that the estimated heritability of bone strength was also moderate (0.26) [9]. However, cartilage research related to pigs is very scarce. The genetic mechanism of cartilage development has yet to be explored.

Therefore, RNA sequencing of the proximal tibial cartilage of Yorkshire piglets in different developmental stages was mainly used in this study. With a transcriptome analysis strategy, the DEGs were screened and verified by studying the developmental regulation mechanism of cartilage and related signal pathways. Revealing the genetic mechanism of cartilage development could help to improve the development of limbs and hooves, reduce pig culling rate and mortality, and reduce the economic loss of farms.

## 2. Materials and Methods

### 2.1. Experiment Material

In this experiment, Yorkshire piglets were used as the main experimental animals. All disposals of the experimental animals were approved by the Scientific Ethic Committee of Huazhong Agricultural University (HZAUSW-2017-008). The selected piglets from the experiment came from Guangxi Yangxiang Co., Ltd. The experimental animals were divided into three developmental stages: Days 1, 14, and 28 of birth. The number of piglets assigned to each group is three. Samples of each age are siblings of the same parity of the same sow. The piglets used in the experiment were all females. The selected sows had no leg disease and received normal feeding, sufficient milk, and purebred Danish Yorkshire sows, and we ensured that the selected sows had the same gestation period. When growing to Day 1, Day 14, or Day 28, respectively, the proximal tibia cartilage was collected from the left hind limb and the muscles and other contaminants were removed. All tissues were collected in 30 min and quickly placed in liquid nitrogen. RNA was extracted from the proximal cartilage of the tibia and stored at −80°C for transcriptome sequencing.

### 2.2. RNA Sequencing

A total amount of 1.5 *μ*g RNA per sample was used as input material for the RNA sample preparations. Sequencing libraries were generated using NEBNext® UltraTM RNA Library Prep Kit for Illumina® (NEB, USA) following manufacturer's recommendations and index codes were added to attribute sequences to each sample. Briefly, mRNA was purified from total RNA using poly-T oligo-attached magnetic beads. Fragmentation was carried out using divalent cations under elevated temperature in NEBNext First Strand Synthesis Reaction Buffer (5X). First strand cDNA was synthesized using random hexamer primer and M-MuLV Reverse Transcriptase (RNaseH-). Second strand cDNA synthesis was subsequently performed using DNA Polymerase I and RNase H. Remaining overhangs were converted into blunt ends via exonuclease/polymerase activities. After adenylylation of 3′ ends of DNA fragments, NEBNext Adaptor with hairpin loop structure was ligated to prepare for hybridization. In order to select cDNA fragments with right length, the library fragments were purified with AMPure XP system (Beckman Coulter, Beverly, USA). Then 3 *μ*l USER Enzyme (NEB, USA) was used with size-selected, adaptor-ligated cDNA at 37°C for 15 min followed by 5 min at 95°C before PCR. Then PCR was performed with Phusion High-Fidelity DNA polymerase, Universal PCR Primers, and Index (X) Primer. At last, products were purified (AMPure XP system) and library quality was assessed on the Agilent Bioanalyzer 2100 system. The clustering of the index-coded samples was performed on a cBot Cluster Generation System using HiSeq 4000 PE Cluster Kit (Illumia) according to the manufacturer's instructions. After cluster generation, the library preparations were sequenced on an Illumina Hiseq 4000 platform and 150 bp paired-end reads were generated.

### 2.3. Bioinformatics Analysis

#### 2.3.1. Quality Control and Sequence Alignment

Sequencing errors, low-quality sequences, sequence junctions, and so forth are common in raw reads. Therefore, it is necessary to perform basic retreatment on the FASTQ sequence file obtained by RNA-seq and filter out the sequence with poor sequencing quality to obtain clean reads. Short sequences were mapped to the pig's reference genome version Sus Scrofa 11.1 using HISAT2 software (version 2.1.0). The mapped reads were sorted by SAMtools (version 1.9). The count was calculated by the featureCounts software (version 1.6.2).

#### 2.3.2. Differential Expression Gene

Differential expression analysis of transcriptome was performed by “DESeq2(version 1.22.2)” R packages, and DEGs were screened, using *P*-adj <0.01 and |FC| > 2 as a DEG requirement.

#### 2.3.3. GO and Pathway Enrichment

Based on the differentially expressed gene data after screening, functional annotations were performed using online metascape software (http://metascape.org/gp/index.html), pathway enrichment with KEGG on the DAVID (https://david.ncifcrf.gov/), and reference of Sus Scrofa used in KEGG.

### 2.4. Validation of DEGs by RT-qPCR

According to the transcriptome sequencing data and bioinformatics analysis results, six DEGs with high values of Fold Change, which are related to bone development and disease, are chosen. And the sequences of genes in pigs were obtained in Ensembl (https://asia.ensembl.org/index.html). Primer design was carried out using Primer Premier 5 (http://www.premierbiosoft.com). The primer sequences are shown in Supplementary [Supplementary-material supplementary-material-1]. *β*-Actin was used as an internal reference, and Quantitative PCR amplification was done using TOYOBO's SYBR Green I real-time PCR Mix, Adopt Roche's LightCycler480 Real-Time PCR Detection System. Gene expression on Day 1 was used as a control group. Statistical analysis method on mRNA level comparisons was *t*-test. The relative expression level of the gene was calculated by the 2^−ΔΔCt^ method and calculated as follows: ΔΔ*C*_t_ = (*C*_t_ Target–*C*_t_*β*-actin) Apiece-(*C*_t_ Target–*C*_t_*β*-actin) Control.

## 3. Results

### 3.1. Transcriptome Data Detection

**Figure 1:**
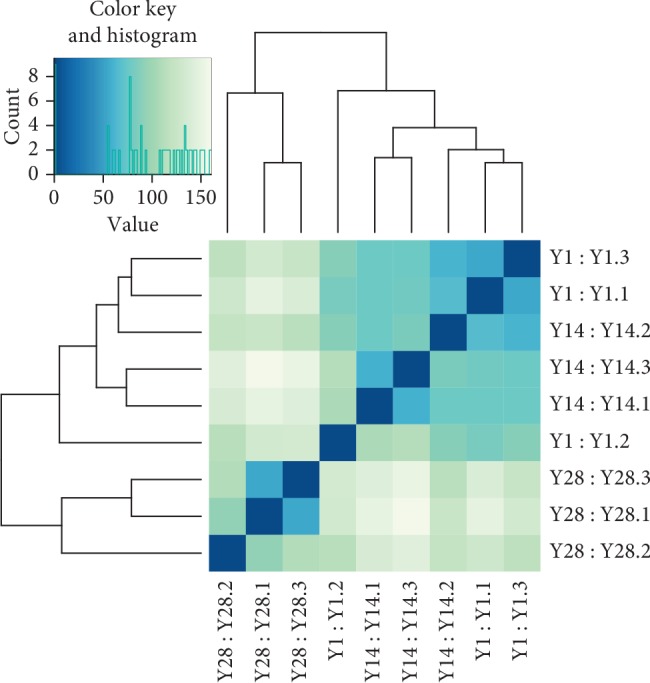
Euclidian distance metric plot. The distance between experimental groups, i.e., time points, is larger than the distance between replicates. The heavier the color was, the closer the sample was.

The clean reads of the obtained proximal tibial cartilage tissue were compared with the reference genome of the pig (Supplementary [Supplementary-material supplementary-material-1]), and the total number of reads (total mapped reads) and the number of reads of the single position alignment (uniquely) were obtained. The above results indicate that the quality of the sequencing data is high, which can be used for subsequent analysis. The R function was used to calculate the distance between the samples.  Figure 1 shows that the 28-day-old sample had the best biological repeatability, followed by the 14-day-old sample, and there is some deviation in the data of 1-day-old piglets; however, the results showed that the sample has a good repeat effect and meets the requirements of further analysis. It can be seen that the difference between the 1-day-old sample and the 14-day-old sample was small, and the 1-day-old and 14-day-old samples were both different from the 28-day-old sample. It can also be inferred that the cartilage development level of piglets at Day 1 and Day 14 was relatively close, and the level of cartilage development of piglets at Day 28 was different.

### 3.2. Differentially Expressed Genes (DEGs)

**Figure 2:**
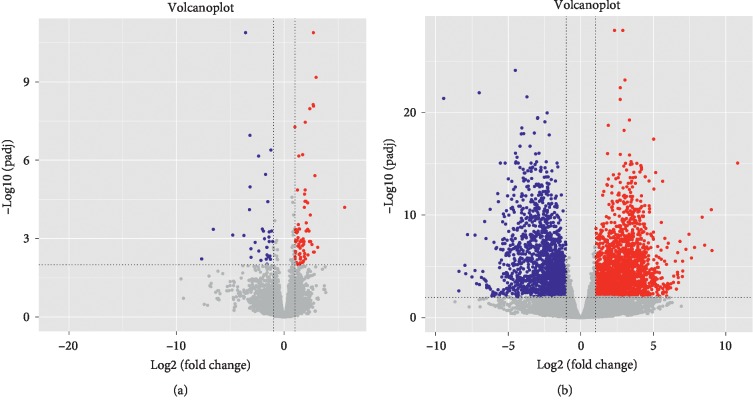
(a) Volcanic maps of 1-day-old and 14-day-old comparison group. (b) Volcanic maps of 14-day-old and 28-day-old comparison group. Blue represents a significantly downregulated gene, and red represents a significantly upregulated gene.

The DEGs of 1-day-old, 14-day-old, and 28-day-old tibia cartilage were compared and divided into a 1-day-old and 14-day-old comparison group and a 14-day-old and 28-day-old comparison group. The most stringent screening conditions were selected by observing the volcano map (Figures 2(a) and 2(b)). Trends in DEGs were observed by making heat maps (Figures [Supplementary-material supplementary-material-1] and [Supplementary-material supplementary-material-1]), and descriptive statistics were performed on the DEGs (Table 1). A total of 108 DEGs were found in the comparison group of Day 1 vs. Day 14, among which 74 DEGs were upregulated and 34 DEGs were downregulated. A total of 3602 DEGs were found in the comparison group of Day 14 vs. Day 28, of which 1897 DEGs were upregulated and 1705 DEGs were downregulated. Obviously, the number of DEGs between Day 14 and Day 28 is much larger than the number of DEGs between Day 1 and Day 14. Therefore, we speculate that Day 14 to Day 28 is a significant change in the cartilage development of the piglets.

### 3.3. GO Analysis of DEGs

**Figure 3:**
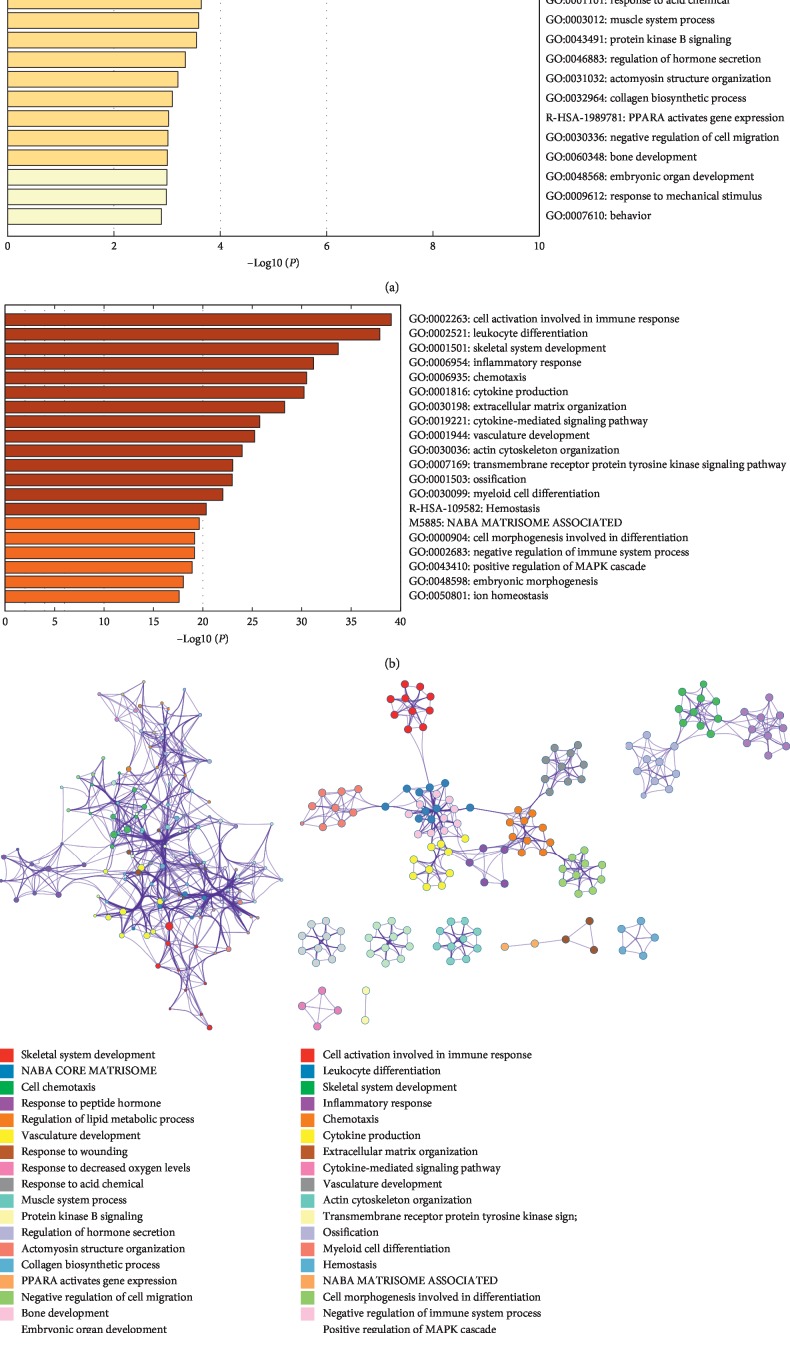
(a, b) Heat map of enriched terms across input gene lists, colored by *P* values: (a) 1-day-old and 14-day-old comparison group's GO classification and (b) 14-day-old and 28-day-old comparison group's GO classification. (c, d) Network of enriched terms: colored by cluster ID, where nodes that share the same cluster ID are typically close to each other: (c) GO term relationship network of 1-day-old and 14-day-old comparison group and (d) GO term relationship network of 14-day-old and 28-day-old comparison group.

GO functional enrichment of DEGs from the two comparison groups is shown in Figure 3(a). GO analysis of DEGs was performed mainly in the skeletal system development, cell chemotaxis, collagen biosynthetic process, bone development, and other GO terms. As shown in Figure 3(b), after the GO analysis of DEGs, the top 20 GO terms were obtained, and the biological processes mainly included skeletal system development, ossification, myeloid cell differentiation, and other GO terms.

To further understand the relationships between the terms, a subset of enriched terms have been selected and rendered as a network plot (Figures 3(c) and 3(d)), where the terms with a similarity >0.3 are connected by edges. We selected the terms with the best *P*values from each of the 20 clusters, with the constraint that there are no more than 15 terms per cluster and no more than 250 terms in total. The network is visualized using metascape, where each node represents an enriched term and was colored first by its cluster ID. We found that the GO terms enriched by the DEGs found in the 1-day-old and 14-day-old comparison group were more closely related, while the GO articles enriched in the 14-day-old and 28-day-old comparison group were relatively independent.

By enriching the GO function of the two comparison groups, we found that more DEGs were aggregated in the comparison of 14-day-old and 28-day-old samples in the GO terms associated with bone development, and there are also more functions related to bone development that are aggregated. We also found DEGs aggregation in ossification, which is an important function of cartilage development. This indicates that more genes related to cartilage development changed from Day 14 to Day 28.

### 3.4. Pathway Enrichment of DEGs

**Figure 4:**
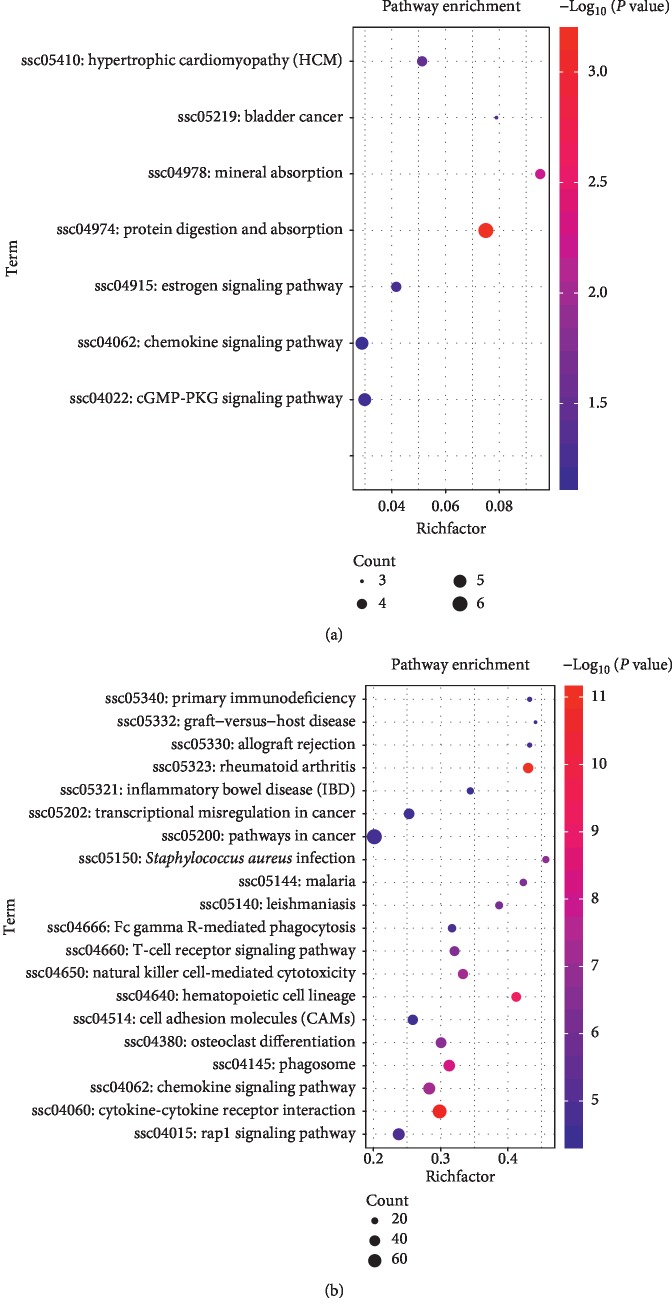
(a) Differential gene pathway enrichment maps of 1-day-old and 14-day-old comparison group. (b) Differential gene pathway enrichment maps of 14-day-old and 28-day-old comparison group. Here, the abscissa is the rich factor, and the ordinate is the enriched path name.

Pathway enrichment of DEGs mapping is shown in Figures 4(a) and 4(b). In 1-day-old and 14-day-old comparison groups, the DEGs enrichment has fewer pathways, so the DEGs with *P*-adj <0.05 are used for pathway enrichment, and the DEGs are mainly enriched in mineral absorption, protein digestion and absorption, and chemokine signaling pathway. In the comparison between the 14-day-old and the 28-day-old groups, the DEGs were mainly enriched in pathways such as rheumatoid arthritis, hematopoietic cell lineage, osteoclast differentiation, and chemokine signaling pathway. The pathway enrichment of DEGs found that from Day 1 to Day 14, the change is more significant for mineral absorption, and from Day 14 to Day 28, it is more significant for osteoclast differentiation. At the same time, we selected one pathway of interest in each of the two comparison groups to indicate the location of gene enrichment as shown in Figures [Supplementary-material supplementary-material-1] and [Supplementary-material supplementary-material-1]. A total of four genes were enriched in the pathway of mineral absorption, in which *MT-2B* and *ICA* were upregulated on Day 14, and *ATP1A2* and *ENSSSCG00000027157* were downregulated on Day 14. A total of 40 genes were enriched in the osteoclast differentiation pathway; with the exception of *AKT3*, *FOS*, *LOC100626904*, *MAPK10*, and *NFATC2*, all other 35 genes were upregulated at 28 days.

### 3.5. Expression Pattern Analysis

**Figure 5:**
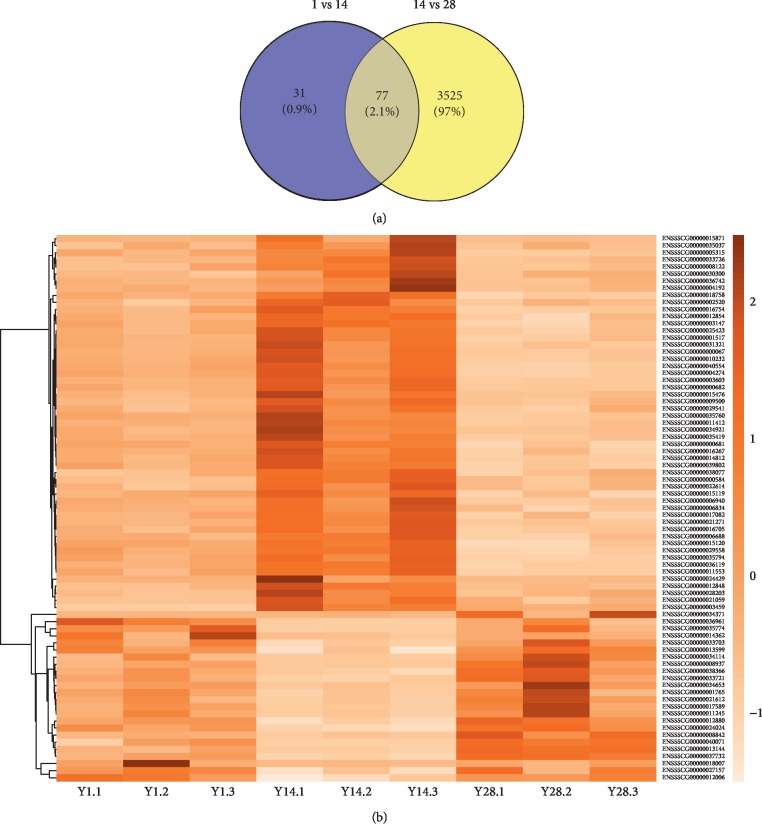
(a) The DEGs Venn diagram of two comparison groups. (b) A heat map of the expression changes of the DEGs shared by the two comparison groups in three periods.

Totally, 3602 DEGs were found by comparison of 14-day-old and 28-day-old groups, far more than the 108 DEGs obtained from comparison between 1-day-old and 14-day-old groups. Thus, compared with Day 1 to Day 14, Day 14 to Day 28 may be a special period of cartilage developmental changes. Therefore, we analyzed the expression patterns of the DEGs shared by the two comparison groups. We found that there are 77 identical DEGs between the two comparison groups (Figure 5(a)). As shown in Figure 5(b), the expression trend of these 77 DEGs is obtained. We can see that the trend of expression of these genes is clearly divided into two groups. The DEGs of the first cluster were relatively at a lower expression level on Day 1 and Day 28 and at a relatively higher expression level on Day 14, and the first cluster has 53 DEGs. There were 24 DEGs aggregated in the second cluster, which was at a relatively high expression level on Days 1 and 28 and a relatively low expression level on Day 14. Then, six candidate genes which were significantly different in at least one comparison (*P*-adj <0.05) and related to cartilage development were selected among the two comparison groups, and the expression trends of the three periods were observed, as shown in [Supplementary-material supplementary-material-1]. It was clearly found that Day 14 was a special period of cartilage development compared to Day 1 and Day 28.

### 3.6. Validation of DEGs by Quantitative PCR (qPCR)

**Figure 6:**
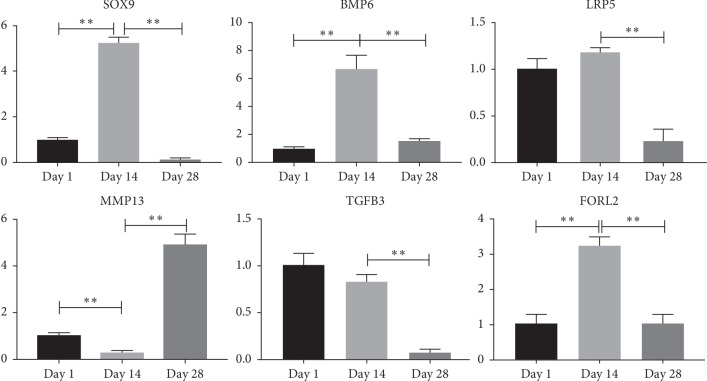
Validation of DEGs by Quantitative PCR (qPCR). Gene expression on Day 1 has used as a control group. Statistical analysis method on mRNA level comparisons was *t*-test, ^*∗*^*P* < 0.05, and ^*∗∗*^*P* < 0.01. Error bars exhibit standard errors.

**Figure 7:**
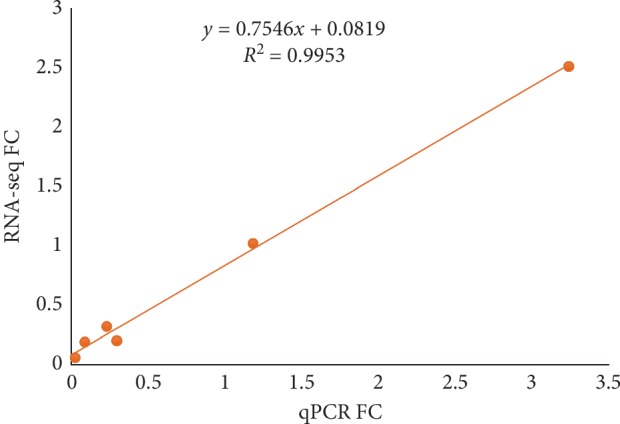
Analysis of fold Change of RNA-seq and qPCR by regression analysis.

Real-time PCRs were performed to verify these six DEGs. The results showed (Figure 6) that *SOX9*, *BMP6*, and *FORL2* were significantly upregulated on Day 14 and significantly downregulated on Day 1 and Day 28. The transcription factor Sox9 is required for the proliferation and differentiation of mesenchymal cells into chondrocytes. And Bmp6 is a member of the bone morphogenetic protein family and plays an important role in chondrogenesis and differentiation. FOLR2 plays a role in the regulation of folate receptors, and folate receptors have a great relationship with macrophages in the synovial membrane of osteoarthritis. The expression level of *LRP5* at Day 14 was higher than that of Day 1, and the expression on Day 28 was significantly lower than that on Day 14. Lrp5 is one of the receptor proteins of the Wnt signaling pathway and can affect changes in bone mass. The expression level of *MMP13* on Day 1 was higher than that on Day 14, and the expression on Day 28 was significantly higher than that on Day 14. *MMP13* is involved in the degradation of collagen matrix during bone and cartilage development. The expression of *TGFB3* on Day 28 was significantly lower than that on other days, and the expression level on Day 14 was lower than that on Day 1. TGF*β* is associated with chondrogenesis and differentiation, and deletion may not affect cartilage formation but dissipate the function of differentiation. The regression analysis results of qPCR and RNA-seq data are shown in Figure 7. The result shows a certain reliability. Comparing the RNA sequencing results with the quantitative PCR experiments (Supplementary [Supplementary-material supplementary-material-1]), it was found that the results of the five genes *SOX9*, *LRP5*, *BMP6*, *MMP13*, and *FOLR2* were consistent, while *TGFB3* was slightly different from the sequencing results.

## 4. Discussion

Pig's limb and foot firmness is a quantitative trait, and genetic factors are among the most important factors. In 2013, Nikkilä et al. found that the leg heritability of pigs was 0.07–0.31 [10]. At the same time, differences in breeds can also lead to differences in heritability. Huang et al. reported that the leg and heritability of Duroc, Landrace, and Yorkshire pigs were 0.23, 0.30, and 0.39, respectively [11]. Although the heritability of these reports varies, it can be shown that the occurrence of leg and foot disease is affected by genetic factors. In 2010, Laenoi et al. used molecular biology experiments to find that MGP (Matrix gla protein) is a potential calcification inhibitor of an extracellular matrix. The mRNA abundance of healthy cartilage MGP is significantly lower than that of rickets [12]. Rangkasenee et al. found that the *KRT8* gene is associated with bone mineral density and content in 310 binary and 298 commercial groups, the *FAF1* gene is associated with articular cartilage disease, and the *PTH1R* gene is significantly associated with the articular cartilage score, and they are related to leg weakness [13].

Based on the results of transcriptome analysis, 108 DEGs were obtained in the 1-day-old and 14-day-old comparison group and 3602 DEGs in the 14-day-old and 28-day-old comparison group. It can be seen from the results that the number of DEGs in the 14-day-old and 28-day-old comparison group is much larger than that in the 1-day-old and 14-day-old comparison group. At the same time, through sample repeatability analysis, we found that the difference between the 1-day-old and 14-day-old samples was small. After GO functional enrichment and KEGG pathway enrichment analysis, the comparison group on Day 1 and Day 14 lacked bone developmental biological processes, such as osteoblast differentiation and osteoclast differentiation, as well as Wnt signaling pathway and BMP signaling pathway. This indicates that there is little difference in the molecular regulation of cartilage development on the day of birth and Day 14; however, after Day 14, DEGs are enriched in skeletal system development, ossification, myeloid cell differentiation, and other GO terms, as well as the osteoclast differentiation pathways. It is indicated that the molecular regulation of cartilage development varies with development time. Through we analyzed the common differential expression patterns in the two comparison groups and the changes in the expression levels of the selected candidate genes during the three periods, we found that Day 14 is in a relatively special time point, and it can even be said to be a turning point. Therefore, based on the above results, we speculate that the process from Day 14 to Day 28 is a process of greater changes in cartilage development, which was not found in previous studies.

Current studies have found that many signal pathways are involved in bone development, such as the Wnt signaling pathway, BMP signaling pathway, TGF*β* signaling pathway, and Hedgehog signaling pathway. The Wnt signaling pathway plays an important role in the development of various organs and tissues, including bone. Its discovery provides new ideas and directions for studying bone development in the future. Wnt/*β*-catenin (Wnt/*β*-catenin) is particularly important for bone biology. Low-density lipoprotein receptor-related protein (LRP5) is an important protein molecule in the Wnt signaling pathway, and DKK1 and SOST are two endogenous inhibitors in the Wnt pathway. *Lrp5* under normal conditions is combined with an endogenous inhibitor. When a missense mutation occurs in Lrp5, it can destroy its own binding to the inhibitor but does not affect the binding to the agonist and thus increases the bone mass, Currently, results in humans [14] and mice [15] have been reported in the literature. A number of studies have reported that this increase in bone mass is primarily a transmission of Wnt/*β*-catenin signal [16, 17]. And SMO in the Hedgehog (Hh) signaling pathway was also found to be differentially expressed during Day 14 vs. Day 28. Hedgehog was originally isolated from the body of Drosophila, and its mutations can make Drosophila embryos develop into hairy cells, which is also known as the hedgehog gene. The transmission of vertebrate Hh signal is mainly through two multichannel transmembrane proteins, Smoothened (Smo) and Patched 1 (Ptch1) [18]. The Hedgehog (Hh) signal is required for the differentiation of mesenchymal stem cells into osteoblasts during embryonic development.

In our study, the DEGs in the comparison group on Days 1 and 14 were mainly enriched in the pathway of mineral absorption. Recent studies have shown that many trace elements, including Zn, Cu, and Fe, are bone-protecting elements that lack the ability to delay bone mass increase during childhood and adolescence and accelerate bone loss at old or postmenopausal age [19]. Various trace elements affect the metabolism and production of bone by regulating the activity of different enzymes in bone metabolism, regulating calcium absorption and excretion, and promoting different pathways, such as osteogenesis and ossification [20, 21]. In 1989, Hall et al. reported that 27 children with reduced blood levels of Mn had ischemic necrotizing bone disease or selective growth disorder, and this Mn deficiency caused ischemic bone disease in chickens [22]. Another study by Staley showed that congenital skeletal malformations in Holstein calves are associated with maternal Mn deficiency [23]. Fe-deficient rats can present with bone mineralization and pathological changes in trabecular bone structure [24, 25] and severe iron deficiency anemia, and rats showed reduced bone matrix formation and decreased bone mineralization [26]. The recommended intake of Mn in the United States is 1.8 to 2.3 mg/d [27]. However, some elements, such as aluminum, cadmium, and lead, are toxic, and these elements can make the quality of bone deteriorate [28]. The DEGs in the comparison group on Days 14 and 28 were mainly enriched in the pathway of osteoclast differentiation. The osteoclast is a tissue-specific macrophage polykaryon created by the differentiation of monocyte/macrophage precursors cells at or near the bone surface [29]. Once formed, bone undergoes a process termed remodeling that involves the breakdown (resorption) and build-up (synthesis) of bone; this occurs in microscale throughout the skeleton. Bone remodeling is the predominant metabolic process regulating bone structure and function during adult life, with the key participant being the osteoclast [30, 31]. Most adult skeletal diseases are due to excess osteoclastic activity, leading to an imbalance in bone remodeling which favors resorption [32]. Such diseases would include osteoporosis, periodontal disease, rheumatoid arthritis, multiple myeloma, and metastatic cancers. The study of this pathway provides a deeper understanding of how diverse physiological and pathophysiological signals exert their effects on the RANK signaling pathway to induce osteoclastogenesis, bone resorption, and skeletal remodeling and so control bone mass [31].

According to the results of DEGs, six differentially large multiples and research-related DEGs were selected, namely, *SOX9*, *LRP5*, *BMP6*, *MMP13*, *TGFβ3*, and *FOLR2*. The transcription factor Sox9 is required for the proliferation and differentiation of mesenchymal cells into chondrocytes. If Sox9 is deleted, cartilage formation is blocked [4], and premature maturation of immature chondrocytes into mast cells takes place [33]. At the same time, Sox9 is thought to directly activate chondrocyte differentiation markers [34], which can induce the expression of Sox5 and Sox6 [5] together with Sox9 to activate the chondrocyte differentiation program [35]. Lrp5, a low-density lipoprotein receptor protein, is one of the Wnt signaling pathway receptors; Lrp5 deletion or missense mutations disrupt binding to inhibitors but do not disrupt binding to agonists, leading to changes in bone mass [36]. Bmp6 is a member of the bone morphogenetic protein family and plays an important role in chondrogenesis and differentiation. When Bmps lacks receptor binding, the expression of *Sox5*, *Sox6*, and *Sox9* is absent in the cartilage anterior condensation of mice and does not produce chondrocytes [37]. TGF*β* is associated with chondrogenesis and differentiation, and deletion may not affect cartilage formation but dissipate the function of differentiation [38]. The role of transforming growth factor beta (TGF*β*) signaling in cartilage has been controversial. Functional studies in cell culture models have shown that TGF*β* can trigger cartilage formation [39]. However, blocking TGF*β* signaling in limbal mesenchymal cells by targeting receptor TGF*β*2 results in a certain effect on chondrocyte differentiation and joint formation but does not seem to affect the initial stage of cartilage [38]. Matrix metalloproteinase 13 (*MMP13*) is secreted by multiple myeloma cells and induces osteoclast active factors produced by multiple myeloma cells. *MMP13* has been implicated in degradation of the collagenous matrices during the development of bone and cartilage [40]. *FOLR2* plays a role in the regulation of folate receptors, and folate receptors have a great relationship with macrophages in the synovial membrane of osteoarthritis [41]. After real-time quantitative PCR in each cartilage tissue, it was consistent with the trend of sequencing results.

## 5. Conclusions

In this study, a transcriptome analysis of the tibial cartilage from Yorkshire pigs with different development stages was performed. Totally, 108 DEGs were found in the 1-day-old and 14-day-old comparison group, and 3360 DEGs were found in the 14-day-old and 28-day-old comparison group as well. These clues showed that the number of DEGs from 14-day-old to 28-day-old group was much larger than that from 1-day-old to 14-day-old group. It is speculated that Day 14 to Day 28 is a critical period of cartilage developmental changes. Furthermore, transcriptome analysis and real-time PCR showed that the Fold Change of *SOX9*, *FOLR2*, *LRP5*, *BMP6*, *MMP13*, and *TGFB3* genes was significant and there are reports related to bone, which was an important candidate gene affecting cartilage phenotype. This study identified candidate genes that affect the development of tibial cartilage, which will compensate for the vacancies in cartilage development-related research. It also inferred the critical period of cartilage development and made an indelible contribution to reveal the genetic mechanism of tibial cartilage development.

## Figures and Tables

**Table 1:** DEGs of tibial cartilage RNA at different ages of piglets.

	Upregulated genes	Downregulated genes
Day 1 vs. Day 14	74	34
Day 14 vs. Day 28	1897	1705

## Data Availability

The data used to support the findings of this study are available from the corresponding author upon request. RNA-seq data from this study have been submitted to the NCBI (SRP181140).

## Supplementary Materials

Supplementary Materials Supplementary Table 1: comparison of tibial cartilage and pig reference genome. Supplementary Table 2: primers for real-time qPCR. Supplementary Table 3: the fold change of gene measuring by qPCR and RNA-seq. Supplementary Figure 1: heat maps of 1-day-old and 14-day-old comparison group. Supplementary Figure 2: heat maps of 14-day-old and 28-day-old comparison group. Supplementary Figure 3: pathway of mineral absorption. Supplementary Figure 4: pathway of osteoclast differentiation. Supplementary Figure 5: the line of relative expression of selected candidate genes in these three periods.
