# Supplementary material for: Identification of mRNAs Related to Tibial Cartilage Development of Yorkshire Piglets
Source: Biomed Res Int. 2019 Nov 5;2019:2365416. doi: 10.1155/2019/2365416 (PMC6875239; doi:10.1155/2019/2365416)
Supplement: Supplementary Materials — Supplementary Table 1: comparison of tibial cartilage and pig reference genome. Supplementary Table 2: primers for real-time qPCR. Supplementary Table 3: the fold change of gene measuring by qPCR and RNA-seq. Supplementary Figure 1: heat maps of 1-day-old and 14-day-old comparison group. Supplementary Figure 2: heat maps of 14-day-old and 28-day-old comparison group. Supplementary Figure 3: pathway of mineral absorption. Supplementary Figure 4: pathway of osteoclast differentiation. Supplementary Figure 5: the line of relative expression of selected candidate genes in these three periods. [file 2365416.f1.docx]

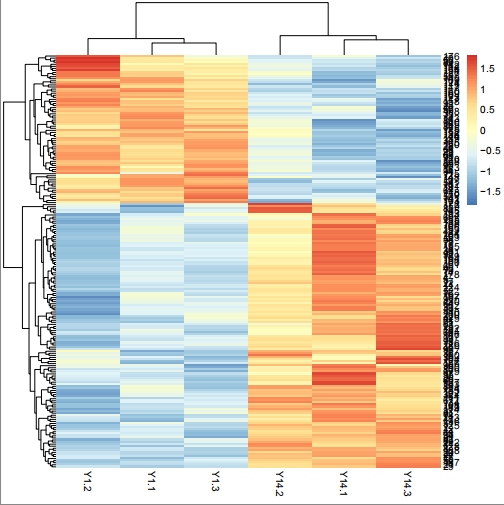


Supplementary Fig.1

heat maps of 1-day-old and 14-day-old comparison group.

one row is a sample, one line is a differential gene, and the color from blue to red represents the low to high expression.


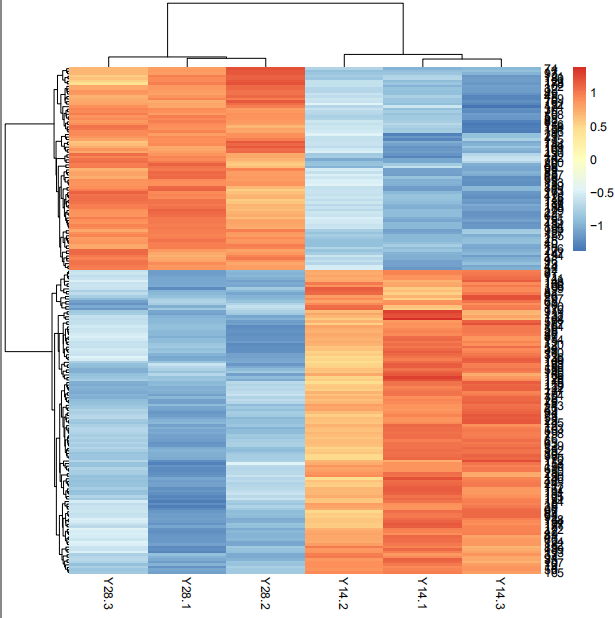


Supplementary Fig.2

heat maps of 14-day-old and 28-day-old comparison group.

one row is a sample, one line is a differential gene, and the color from blue to red represents the low to high expression.


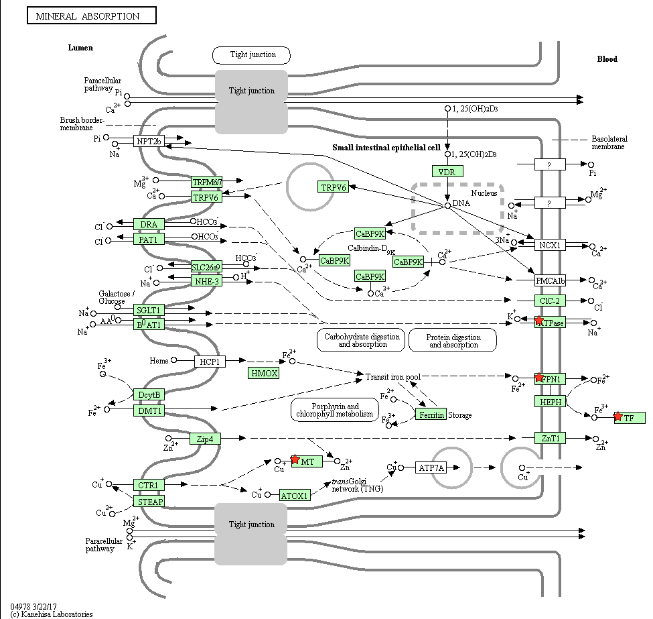


Supplementary Fig.3


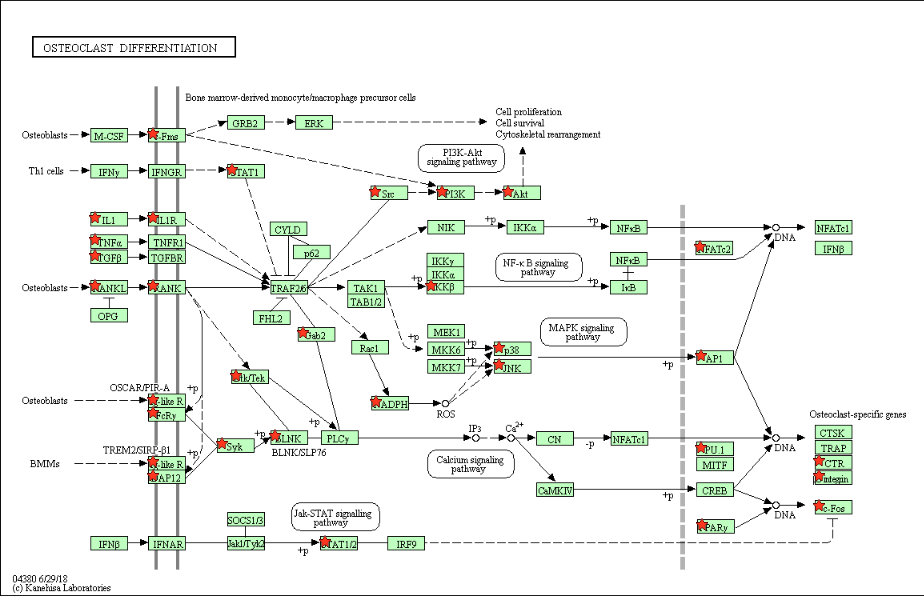


Supplementary Fig.4

Supplementary Fig.3 and 4 are schematic representations of the location of the differential gene in the major pathway; the red star is the differential gene obtained.


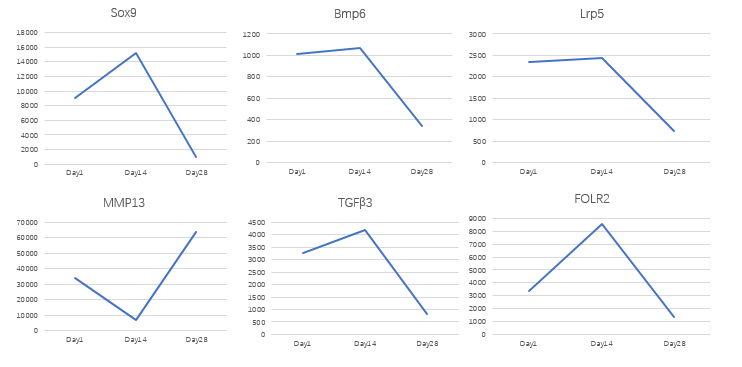


Supplementary Fig.5

the line of relative expression of selected candidate genes in these three periods.

**Supplementary Table 1** **Comparison of tibial cartilage and pig reference genome**

| Sample name | Total reads | Total mapped | Uniquely mapped | Multiple mapped |
| --- | --- | --- | --- | --- |
| Y1-1Jruanjin | 24725778 | 23918968（96.74%） | 17033508（71.21%） | 6885460（28.79%） |
| Y1-2Jruanjin | 26923411 | 25614361（95.14%） | 17975317（70.18%） | 7639044（29.82%） |
| Y1-3Jruanjin | 24501975 | 23384527（95.44%） | 16047855（68.63%） | 7336672（31.37%） |
| Y14-1Jruanjin | 23887133 | 23048246（96.49%） | 13776440（59.77%） | 9271806（40.23%） |
| Y14-2Jruanjin | 29579461 | 28321675（95.75%） | 17365299(61.31%) | 10956376(38.69%) |
| Y14-3Jruanjin | 20047560 | 19230682（95.93%） | 11147427(57.97%) | 8083255(42.03%) |
| Y28-1Jruanjin | 26309248 | 25460417（96.77%） | 18266854(71.75%) | 7193563(28.25%) |
| Y28-2Jruanjin | 24661459 | 23666490（95.97%） | 16632574(70.28%) | 7033916(29.72%) |
| Y28-3Jruanjin | 23510113 | 22656998（96.37%） | 15913995（70.24%） | 6743003（29.76%） |

**Supplementary Table 2** **Primers for real-time qPCR**

| Primer | Sequence of primers (5’-3’) | Tm(°C) | Size (bp) |
| --- | --- | --- | --- |
| Sox9-F | CCAGCGAACGCACATCAAG | 60 | 144 |
| Sox9-R | TACTGCGAGCGGGTGATGG |  |  |
| Lrp5-F | GCTCCATCCACGCCTGTAA | 60 | 112 |
| Lrp5-R | AAGTAAGGCTGCCGCTCTG |  |  |
| Bmp6-F | AGGACGGGCTCAGCATCA | 60 | 374 |
| Bmp6-R | GTGGTTGGTGGCGTTCAT |  |  |
| TGFβ3-F | CCTTTACAACAGCACCCG | 54 | 426 |
| TGFβ3-R | AAGAGCCATTCACGVAVA |  |  |
| MMP13-F | CACCCGTGACCTTATCTT | 54 | 326 |
| MMP13-R | ACTGTATGGGACCGTTGA |  |  |
| FOLR2-F  FOLR2-R | TGGACTGGTGGGAAGACT  CCTCGCTACCTCCTCGTT | 58 | 263 |
| β-actin-F | CCAGGTCATCACCATCGG | 60 | 158 |
| β-actin-R | CCGTGTTGGCGTAGAGGT |  |  |

**Note: F refers to Forward primer; R refers to Reverse primer.**

**Supplementary Table 3 The Fold Change of gene measuring by qPCR and RNA-seq**

|  |  | qPCR |  |  | RNA-seq |  |
| --- | --- | --- | --- | --- | --- | --- |
|  | Day1 | Day14 | Day28 | Day1 | Day14 | Day28 |
| TGFB3 | 1 | 0.827564 | 0.072949 | 1 | 1.259499 | 0.242045 |
| FORL2 | 1 | 3.24096 | 1.029801 | 1 | 2.513772 | 0.400396 |
| BMP6 | 1 | 6.71618 | 1.556295 | 1 | 1.03929 | 0.334813 |
| MMP13 | 1 | 0.296295 | 4.934489 | 1 | 0.203253 | 1.826286 |
| LRP5 | 1 | 1.183517 | 0.236586 | 1 | 1.022775 | 0.308039 |
| SOX9 | 1 | 5.269311 | 0.136799 | 1 | 1.660929 | 0.100473 |
